# Advancing sustainable nursing leadership: the interplay of green absorptive capacity, intellectual capital, and knowledge management among nursing managers

**DOI:** 10.1186/s12912-025-03769-4

**Published:** 2025-09-01

**Authors:** Heba Sobhy Mohamed, Ahmed Gamal Ghazaly Radwan, Asmaa Saber Ahmed Ahmed Elborai, Alaa Eldin Moustafa Hamed, Abeer Moustafa Barakat, Mohammed Adel Abd Elhafeez Elbakry, Zienab Ibrahem Ismael

**Affiliations:** 1 Nursing Administration, Faculty of Nursing, Zagazig University, Zagazig, Egypt; 2 Business Administration, Faculty of Commerce, Benha University, Benha, Egypt; 3 Nursing Administration, Faculty of Nursing, Ain Shams University, Cairo, Egypt; 4 Psychiatric/Mental Health Nursing, Faculty of Nursing, Helwan University, Cairo, Egypt; 5 Maternal and Newborn Health Nursing, Faculty of Nursing, Helwan University, Cairo, Egypt; 6 Nursing Administration, Faculty of Nursing, Benha University, Benha, Egypt

**Keywords:** Green absorptive capacity, Green intellectual capital, Green knowledge management, Sustainable performance, Nursing leadership

## Abstract

**Background:**

Achieving sustainable performance in healthcare organizations has become a pressing necessity, driven by environmental challenges and the imperative for enhanced efficiency. Within nursing leadership, the integration of green absorptive capacity, green intellectual capital, and green knowledge management has emerged as a strategic approach to fostering sustainability. However, empirical research on the interplay of these green competencies in the nursing sector remains limited.

**Aim:**

This study aims to examine the mediating role of green knowledge management (GKM) in the relationships between green absorptive capacity, green intellectual capital, and sustainable performance among nursing managers. It explores how GKM processes translate environmental competencies into sustainable leadership outcomes.

**Methods:**

A cross-sectional descriptive correlational study was conducted among 207 nursing managers at Zagazig University Hospitals, Egypt. Data was collected using five validated instruments, including the Green Absorptive Capacity Questionnaire, Green Intellectual Capital Questionnaire, Sustainable Performance Questionnaire, and Green Knowledge Management Scale. Structural equation modeling and path analysis were performed to test the study hypotheses.

**Results:**

The findings revealed significant positive associations between green absorptive capacity (β = 0.123, *p* < 0.001), green intellectual capital (β = 0.064, *p* = 0.016), and sustainable performance among nursing managers. Green knowledge management exhibited the strongest direct effect on sustainable performance (β = 0.727, *p* < 0.001). Additionally, green knowledge management significantly moderated the relationships between green absorptive capacity and sustainable performance (β = 0.256, *p* < 0.001) as well as between green intellectual capital and sustainable performance (β = 0.359, *p* < 0.001). These results underscore the pivotal role of knowledge-driven sustainability strategies in nursing leadership.

**Conclusion:**

This study highlights the importance of integrating green absorptive capacity, green intellectual capital, and green knowledge management into nursing leadership to enhance sustainability outcomes. The findings provide empirical evidence for the need to embed green competencies within nursing management, advocating for policies and training programs that reinforce environmental responsibility in healthcare institutions.

**Clinical trial number:**

Not applicable.

## Background

The necessity for sustainable performance in healthcare organizations has become increasingly urgent due to environmental challenges and the need for improved efficiency [1]. Organizations that successfully implement green strategies not only minimize their negative environmental impact but also enhance their long-term competitiveness and sustainability [2]. In the healthcare sector, sustainable performance (SP) refers to an interconnected system of practices aimed at managing, restoring, and improving human health while considering environmental, economic, and social factors. It promotes harmony between healthcare systems, the natural environment, and society [3, 4]. For this study, sustainable performance is used as a proxy for sustainable nursing leadership, as it reflects the measurable outcomes of leadership practices that align with environmental responsibility and long-term healthcare goals.

Nurses play a critical role in maintaining a safe and sustainable healthcare environment for current and future generations [5]. Given the ethical responsibility to integrate a health-in-all-policies approach, healthcare organizations must embrace sustainable strategies and foster environmental awareness among nursing professionals [6]. Sustainable performance in nursing can be improved through knowledge acquisition, implementation of green initiatives, and collaboration with stakeholders [7, 8].

Green Knowledge Management (GKM) is increasingly recognized as a vital strategy for healthcare organizations addressing environmental challenges and striving for Sustainable Development Goals (SDGs) [9, 10]. In nursing practice, GKM can be applied through initiatives such as personnel training on waste segregation protocols, development of environmental safety guidelines and interprofessional knowledge sharing to encourage eco-friendly clinical behaviors. It facilitates the adoption of sustainable practices through effective knowledge acquisition and integration, enabling healthcare institutions to implement eco-friendly initiatives such as waste reduction, responsible water usage, and safer chemical alternatives [8, 11]. Additionally, Green Intellectual Capital (GIC), which encompasses intangible assets related to environmental sustainability plays a crucial role in promoting these initiatives [12, 13]. In a nursing context, GIC becomes tangible through staff-led environmental audits, participation in green innovation projects, and collaboration with external partners on health-related environmental advocacy. By cultivating Green Absorptive Capacity (GAC), nursing managers can significantly enhance hospital practices in waste management, energy conservation, and the reduction of harmful chemicals, ultimately fostering a sustainable healthcare environment [14–16].

GIC comprises green human capital (GHC), green structural capital (GSC), and green relational capital (GRC). GHC refers to the ecological knowledge, skills, abilities, and creativity of employees that contribute to sustainability [17]. For example, in clinical nursing, this includes competencies in safely handling hazardous materials, conserving energy and water during care procedures, and suggesting low-waste alternatives in patient care. GSC includes an organization’s policies, culture, knowledge systems, and innovations related to environmental sustainability [18]. This might involve protocols for purchasing green electronic systems for tracking resource usage, or institution-wide campaigns to reduce carbon emissions in healthcare delivery. Lastly, GRC encompasses the relationships between healthcare organizations and external stakeholders, including suppliers, policymakers, and the community that influence sustainable practices [19].

A crucial mechanism linking GIC to improved environmental performance is green absorptive capacity (GAC), an organization’s ability to identify, acquire, assimilate, and apply external knowledge for sustainable practices [20, 21]. In nursing environments, GAC is reflected in ongoing professional development programs on environmental topics, cross-unit collaboration on sustainable procedures, and the inclusion of sustainability metrics in nursing management evaluations. GAC strengthens an organization’s ability to leverage expertise, enhance green skills, and integrate sustainability-oriented innovations [22].

Despite a growing body of research examining the roles of GKM and intellectual capital in various sectors [23, 24], their specific influence within the healthcare domain especially from the perspective of nursing managers remains underexplored. Previous studies have mainly addressed green human resource management [25] or the dimensions of intellectual capital in hospital settings [26]. while only a few have considered knowledge management’s impact on healthcare sustainability [27, 28]. Recent findings underscore that green intellectual capital significantly contributes to competitive advantage and organizational sustainability in nursing and healthcare leadership contexts [29, 30]. Additionally, growing emphasis has been placed on the integration of environmental knowledge into nursing practice to foster eco-conscious leadership and improve sustainability performance [5, 31]. However, the mediating role of GKM in the relationships between GIC, GAC, and sustainable performance within nursing leadership is still theoretically and empirically unexamined.

This study is grounded in the Resource-Based View (RBV) and Knowledge-Based View (KBV) of the firm. According to RBV, unique, valuable, rare, and inimitable resources such as GIC and GAC provide a competitive advantage and drive superior performance [32, 33]. In nursing leadership, GIC represents the strategic environmental knowledge, skills and relational networks that can differentiate healthcare organizations in sustainability outcomes, while GAC reflects the ability to transform external environmental knowledge into actionable nursing practices. KBV extends this logic by emphasizing that knowledge is the most strategically significant resource for organizations. Within this perspective, GKM becomes the critical process through which the knowledge embedded in GIC and accessed via GAC is captured, shared and applied to achieve SP. Thus, RBV explains what strategic green resources lead to performance (GIC, GAC), whereas KBV explains how these resources are converted into sustainable outcomes through effective knowledge management (GKM).

Drawing from RBV, it is expected that both GAC and GIC will have positive direct effects on SP (H1, H2) because they embody rare and valuable capabilities that can yield long-term sustainable advantages in nursing leadership. Guided by KBV, GKM is hypothesized to directly enhance SP (H3) and mediate the effects of GIC and GAC on SP (H4, H5), as it operationalizes the transformation of environmental knowledge into concrete sustainable practices. This dual-theory integration provides a robust explanation for the proposed conceptual model, where RBV underpins the possession of strategic green resources and KBV underscores the processes that leverage these resources for sustainable outcomes.

**Fig. 1:**
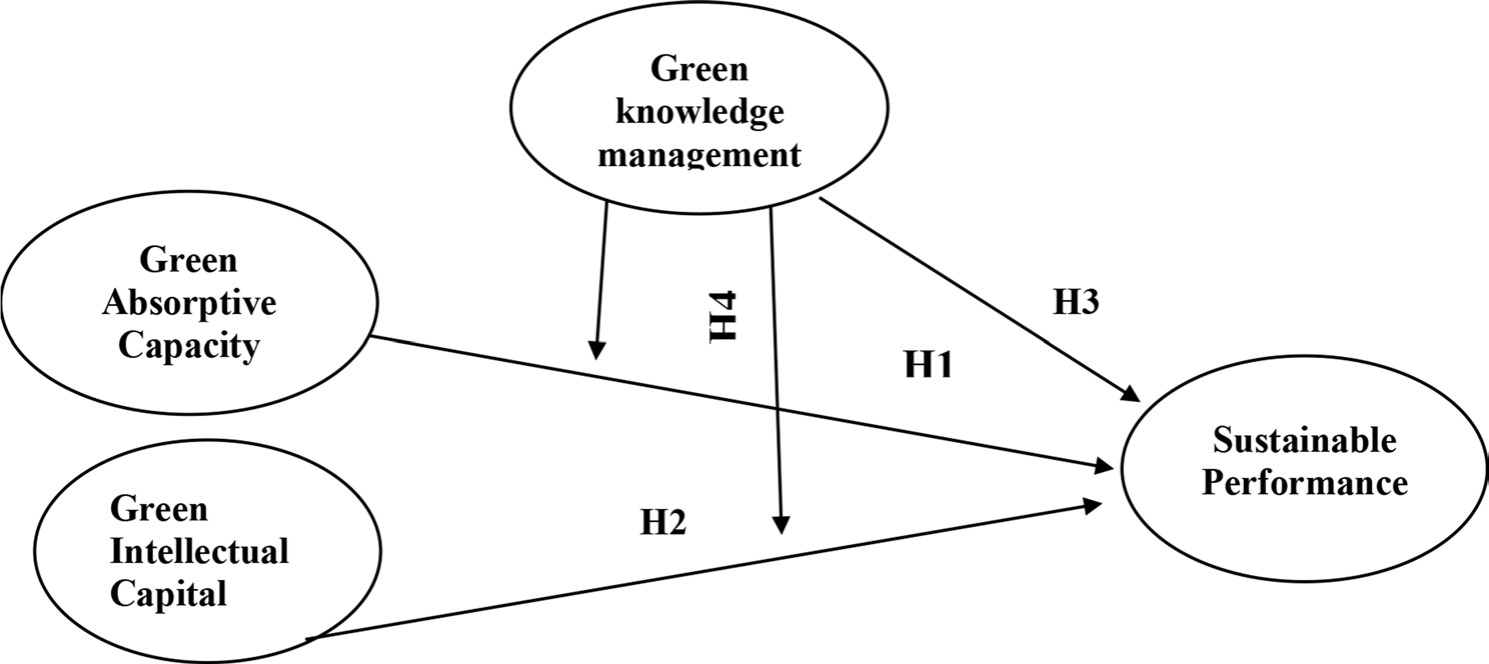
Conceptual framework

To address this critical gap, the present study investigates the direct effects of green absorptive capacity (GAC) and green intellectual capital (GIC) on sustainable performance (SP), as well as the mediating role of green knowledge management (GKM) in these relationships among nursing managers. In doing so, the study advances both theoretical understanding and practical strategies for promoting environmentally responsible leadership in healthcare settings. Accordingly, a conceptual model (Fig. 1) is proposed, illustrating the hypothesized relationships among GIC, GAC, GKM, and SP. The model posits that GKM functions as a mediator between both GIC and GAC with SP. This framework is grounded in the assumption that green-related knowledge processes enhance the strategic value of environmental resources by amplifying their influence on organizational outcomes, an approach supported by emerging empirical evidence in green healthcare management [34]. Therefore, the objective of this study is to examine the direct effects of GAC and GIC on sustainable performance and the mediating role of GKM in these relationships. Based on this conceptual foundation, the framework hypothesizes the following hypotheses:

- **H1**: Green absorptive capacity (GAC) is positively and significantly related to nursing managers’ sustainable performance.
- **H2**: Green intellectual capital (GIC) is positively and significantly related to nursing managers’ sustainable performance.
- **H3**: Green knowledge management (GKM) is positively and significantly related to nursing managers’ sustainable performance.
- **H4**: Green knowledge management (GKM) significantly mediates the relationship between green intellectual capital (GIC) and nursing managers’ sustainable performance.
- **H5**: Green knowledge management (GKM) significantly mediates the relationship between green absorptive capacity (GAC) and nursing managers’ sustainable performance.

## Methods

### Aim of the study

This study aims to examine the mediating role of green knowledge management (GKM) in the relationships between green absorptive capacity, green intellectual capital and sustainable performance among nursing managers. It explores how GKM processes translate environmental competencies into sustainable leadership outcomes.

### Setting and study design

This cross-sectional descriptive correlational study was conducted at Zagazig University Hospitals, a major healthcare network affiliated with Zagazig University in Egypt. The hospital system includes various specialty units, providing a comprehensive environment for examining leadership and sustainability practices among nursing managers.

### Sampling and participants

A total of 207 nursing managers were recruited using a convenience sampling approach, which was deemed appropriate due to the practical constraints of accessing a dispersed population of managers within the selected hospitals. This non-probability method allowed for the inclusion of participants who met the predefined eligibility criteria and were available during the data collection period. Eligible participants had a minimum of three years of experience in the study setting, were present at the time of data collection, and provided informed consent to participate.

The required sample size was estimated using the G*Power software (version 3.1.9.7), applying an a priori power analysis for an F test within the context of linear multiple regression (fixed model, R² deviation from zero). The following parameters were used: a medium effect size (f² = 0.15), an alpha error probability of 0.05, power (1 − β) of 0.95, and 5 predictors. Based on these inputs, the minimum required sample size was 138 participants. Therefore, the final sample of 207 exceeded the minimum requirement, ensuring adequate statistical power. The selected nursing managers represented diverse educational backgrounds, including Diploma Nurses, graduates of the Technical Institute of Nursing, bachelor’s degree holders in nursing, and those with postgraduate nursing education. They were employed across various departments within the participating hospitals.

### Study instruments

Five validated instruments were used to collect data for this study: the Nursing Managers’ Sociodemographic Characteristics Questionnaire, Green Absorptive Capacity Questionnaire, Green Intellectual Capital Questionnaire, Sustainable Performance Questionnaire, and Green Knowledge Management Scale. These instruments were administered during face-to-face structured interviews and presented in the participants’ native language (Arabic).

The researchers employed both the committee and back-translation approaches to translate scales originally developed in English [35, 36]. The translation committee comprised four members who independently and concurrently translated the scales: two nursing professors from Egypt, a nurse proficient in English with experience in healthcare settings, and a native English-speaking educator. After the initial translation, a back-translation approach was applied, where the translated versions were independently re-translated into English by two bilingual experts. The final versions of each scale were reviewed and unanimously approved by all committee members.

To ensure validity and cultural relevance in the context of sustainable nursing leadership, the instruments were evaluated by a panel of experts in nursing leadership, green management practices, and healthcare sustainability. This expert panel included nursing management professors, specialists in green healthcare practices, and experts in knowledge management within healthcare settings. Their input helped refine and adapt several questions to better reflect the research’s focus on green absorptive capacity, intellectual capital, and knowledge management. A pilot study involving 21 nursing managers, a sample size appropriate to the final sample of 207 nursing managers, further validated the questionnaire. This pilot study ensured that the instruments were clear and relevant for the target population before they were distributed to nursing managers across various healthcare institutions.

This research instrument consists of five primary sections, with the first section focusing on nursing managers’ sociodemographic characteristics and educational information. Developed by the researchers in their native language (Arabic), this tool captures key personal and professional attributes of the participating nursing managers. It includes variables such as age, gender, education level, job position, residence, marital status, years of experience, monthly income, department, and hospital type. Each item was carefully selected to provide a comprehensive demographic and professional profile of the participants. This structured approach ensures high-quality data collection, enhances the study’s contextual understanding, and supports more rigorous analyses in future research.

The second section employed the Green Absorptive Capacity Questionnaire developed by Begum et al. (2023) [20], a validated instrument designed to assess nursing professionals’ ability to acquire, assimilate, transform, and apply environmental knowledge in healthcare settings. This tool plays a critical role in evaluating the integration of green competencies into nursing practice, thereby supporting sustainable healthcare delivery. The questionnaire consists of 16 items distributed across four dimensions, including awareness, knowledge assimilation, and the practical application of sustainable practices. Nursing managers responded using a five-point Likert scale (1 = “strongly disagree” to 5 = “strongly agree”), with dimension scores calculated by averaging item responses. This approach offers a robust assessment of the extent to which green practices are institutionalized in nursing. The tool’s reliability is well-established, with Marzouk and El Ebrashi (2024) reporting a high Cronbach’s alpha of 0.891, reinforcing its reliability for research on green initiatives and sustainability gaps in healthcare [37]. Our study assessed the reliability of the tool and reported good reliability with Cronbach’s alpha was (α = 0.834).

The third part of the research instrument was the Green Intellectual Capital Questionnaire (GICQ), developed by Edyta Bombiak in 2020 [38]. This tool assesses the integration of Green Intellectual Capital (GIC) within nursing, evaluating sustainability practices through three key components: Green Human Capital, Green Organizational Capital, and Green Relational Capital. The questionnaire consists of 30 items rated on a five-point Likert scale, ranging from 1 (“Strongly Disagree”) to 5 (“Strongly Agree”). Scores were calculated by averaging the responses for each dimension, yielding a total score range of 30 to 150. The reliability of the tool has been supported in previous studies. Khaled et al. (2023) reported Cronbach’s alpha of 0.93, indicating excellent internal consistency [39]. Similarly, Atalla et al. (2024) found the scale to be highly reliable, with a Cronbach’s alpha of 0.92 for the overall questionnaire [29]. In the present study, the reliability of the scale was further assessed, yielding a Cronbach’s alpha of 0.929, confirming its strong internal consistency.

The fourth part of the research instrument was the Sustainable Performance Questionnaire (SPQ), developed by Mousa & Othman (2020) [25]. This tool assesses the integration of Green Human Resource Management (GHRM) practices within nursing, evaluating sustainable performance across three key dimensions: Environmental Performance (EP), Economic Performance (EPc), and Social Performance (SP). The questionnaire consists of 15 items, rated on a five-point Likert scale ranging from 1 (“Not at all”) to 5 (“A very significant extent”). Scores were calculated by averaging responses for each dimension, yielding a total score range of 15 to 75. The reliability of the tool has been supported in previous studies. Al Issa et al. (2023) reported a Cronbach’s alpha of 0.79 [2], while Duque-Uribe et al. (2024) found a higher reliability of 0.866 [40]. In the present study, the scale demonstrated strong internal consistency, yielding a Cronbach’s alpha of 0.888, confirming its reliability in assessing sustainable performance in nursing settings.

The fifth section utilized the Green Knowledge Management (GKM) Scale (Yu et al. 2022) [41]. It is a validated instrument designed to measure the adoption of sustainability-oriented knowledge management practices in nursing. The scale evaluates five core dimensions: Green Knowledge Acquisition (GKA), Green Knowledge Sharing (GKS), Green Knowledge Storage (GKT), Green Knowledge Application (GKAp), and Green Knowledge Creation (GKC). Comprising 27 items, responses were recorded on a seven-point Likert scale (1 = “Strongly Disagree” to 7 = “Strongly Agree”). Dimension scores were derived by averaging item responses, with total scores ranging from 27 to 189. Prior studies have demonstrated the scale’s high reliability, with Sahoo et al. (2023) and Wang et al. (2022) reporting Cronbach’s alpha values of 0.913 and 0.901, respectively [8, 10]. In the current study, reliability was further confirmed, yielding a Cronbach’s alpha of 0.92, reinforcing the tool’s robust internal consistency for assessing green knowledge management in healthcare contexts.

### Procedures

Ethical approval for this study was obtained from the Research Ethics Committee (REC) of the Faculty of Nursing, Zagazig University, Egypt (Approval ID: ZU.Nur.REC#:109) in September 2024. Official permissions were also received from relevant hospital authorities to facilitate data collection. Prior to participation, all nursing managers were informed about the study’s objectives and their rights, including the voluntary nature of participation, confidentiality assurances, and the freedom to withdraw at any stage without repercussions. A pilot study was conducted with 20 randomly selected nursing managers to assess the clarity, feasibility and time burden of the questionnaire tools; no modifications were needed based on pilot results. The main data collection phase took place over one month starting in September 2024, during which researchers met with participants three times weekly in private, distraction-free settings within their respective hospitals. All questionnaires were administered face-to-face using printed forms to ensure consistency. To preserve data integrity and participant anonymity, responses were coded and no identifying information was recorded. Questionnaires were reviewed immediately upon completion to address the missing data in real time. Participants were thanked for their time and contributions, reinforcing respect for their role in the research process.

### Ethical consideration

Ethical approval for conducting this study was obtained from the Research Ethics Committee (REC) of the College of Nursing, Zagazig University, Egypt (Approval ID: ZU.Nur.REC#:109) in September 2024. Prior to participation, the study objectives were thoroughly explained to all participants, emphasizing that the collected data would be used solely for research purposes. Participants were informed of their right to voluntary participation, including the option to decline or withdraw from the study at any stage without facing any negative consequences.

To uphold confidentiality and data protection, all responses were anonymized and securely stored, ensuring that the data remained accessible only to the research team. Informed written consent was obtained from nursing managers who agreed to participate, reinforcing their understanding of the study’s aims and ethical considerations. All study procedures adhered to the ethical guidelines of the Declaration of Helsinki and its subsequent amendments, ensuring the rights, dignity, and welfare of participants were safeguarded throughout the research process.

### Statistical analysis

Data was analyzed using SPSS version 26 (IBM, USA) and AMOS version 24. Prior to conducting inferential analyses, the Shapiro–Wilk test was applied to assess the normality of all study variables. The results confirmed normal distribution for all major constructs (*p* > 0.05), indicating the appropriateness of parametric techniques. No extreme outliers were detected based on standardized z-scores (± 3.29), boxplots, and Mahalanobis distance (*p* < 0.001). Descriptive statistics (mean, standard deviation, frequencies, and percentages) were used to summarize participant characteristics and scale scores. Pearson’s correlation coefficients were calculated to examine bivariate associations. Multiple linear regression analysis was conducted to identify predictors of the outcome variable. Cronbach’s alpha coefficients were used to determine the internal consistency of each instrument. To assess direct and indirect effects among constructs, path analysis was performed using AMOS, which is appropriate for modeling latent variable relationships and estimating structural paths. For mediation testing, Hayes’ PROCESS macro for SPSS (Model 4) was employed to evaluate the mediating role of green knowledge management. Model fit was evaluated using multiple indices: CMIN/DF (< 3), CFI (> 0.90), GFI (> 0.80), TLI (> 0.90), RMSEA (< 0.08), and RMR (< 0.05), with interpretation guided by recommended SEM thresholds. A p-value ≤ 0.05 was considered statistically significant.

## Results

Table 1 presents the demographic characteristics of the study participants, highlighting a predominantly female (56.04%) and married (73.43%) workforce with a mean age of 40.48 ± 7.79 years. The majority held a bachelor’s degree (72.98%) and had 5 to 25 years of experience (67.14%), indicating substantial clinical exposure. A noTable 80.83% reported an unsatisfactory income, and most (69.08%) resided in urban areas. Head nurses constituted the largest job category (80.7%). These findings provide essential context for understanding the professional and socioeconomic factors that may influence nursing practice and workplace dynamics.

**Table 1:** Descriptive statistics of study variables (*n* = 207)

Personal data	Categories	No	%
Age	25-<35	58	28.02
	35-<45	81	39.13
	*≥* 45	68	32.85
	**Mean** ± SD	40.48 ± 7.79	
Gender	Male	91	43.96
	Female	116	56.04
Marital status	Single	37	17.87
	Married	152	73.43
	Divorced/Widow	18	8.70
Educational level	Technical Institute of Nursing	37	17.87
	Bachelor’s degree in nursing	151	72.95
	Postgraduate education	19	9.18
Years of experience	5 to < 15	68	32.85
	15 to < 25	95	45.89
	≥ 25	44	21.26
Monthly Income	Satisfactory	82	39.61
	Unsatisfactory	125	60.93
Residence	Rural	64	30.92
	Urban	143	69.08
Job Position	Head Nurse	167	80.7%
	Nursing supervisor	24	11.6%
	Assistant nursing director	11	5.3%
	Nursing director	5	2.4%
Hospital Type			
	General Medicine Hospital	37	17.87
	Specialized Medicine Hospital	31	14.98
	Pediatrics Hospital	25	12.08
	Emergency (Istekbal) Hospital	21	10.14
	New Surgery / Assalam Hospital	37	17.87
	Tumor & Oncology Hospital	17	8.21
	Accidents & Trauma Hospital	21	10.14
	Economic Treatment Hospital	18	8.70
Hospital Unit	Medical Ward	31	18.56
	Surgical Ward	28	16.77
	Intensive Care Unit (ICU)	23	13.77
	Emergency Unit	20	11.98
	Pediatric Unit	18	10.78
	Obstetrics & Gynecology Unit	16	9.58
	Cardiac/Chest Unit	13	7.78
	Outpatient/Specialty Clinics	12	7.19
	Others	6	3.59

Table 2 presents the descriptive statistics and correlation matrix for the study variables. The results indicate that Green Absorptive Capacity (M = 3.82, SD = 0.98) and Green Intellectual Capital (M = 3.76, SD = 0.89) are positively correlated (*r* = 0.638, *p* < 0.01), suggesting a strong association between organizational knowledge absorption and intellectual capital. Sustainable Performance (M = 4.07, SD = 0.66) shows significant positive correlations with both Green Absorptive Capacity (*r* = 0.697, *p* < 0.01) and Green Intellectual Capital (*r* = 0.474, *p* < 0.01), highlighting their contribution to sustainability outcomes. Additionally, Green Knowledge Management (M = 4.05, SD = 0.67) is significantly associated with all study variables, with the highest correlation observed with Sustainable Performance (*r* = 0.603, *p* < 0.01). These findings underscore the interconnected role of green competencies in enhancing sustainability within healthcare settings.

**Table 2:** Reliability and validity assessment of measurement model (*n* = 207)

variables	Mean	Std. Deviation		GAC	GIC	SP
Green Absorptive Capacity	3.82	0.98	r	1		
			p			
Green intellectual Capital	3.76	0.89	r	0.638^**^	1	
			p	0.000		
Sustainable performance	4.07	0.66	r	0.697^**^	0.474^**^	1
			p	0.000	0.000	
Green knowledge Management	4.05	0.67	r	0.544^**^	0.529^**^	0.603^**^
			p	0.000	0.000	0.000

** Correlation is significant at the 0.01 level

Table 3 presents the reliability, validity, and model fit indicators for the study constructs. The composite reliability (CR) values range from 0.801 to 0.955, exceeding the 0.70 threshold, confirming internal consistency. The average variance extracted (AVE) values are generally above 0.50, supporting convergent validity. Model fit indices indicate an acceptable fit, with Comparative Fit Index (CFI) values ranging from 0.842 to 0.969 and Goodness-of-Fit Index (GFI) values above 0.70. Root Mean Square Error of Approximation (RMSEA) values remain within acceptable limits, reinforcing the model’s robustness. These results validate the measurement model, ensuring reliability and construct validity in assessing green competencies and sustainability-related factors in nursing practice.

**Table 3:** Correlation matrix of key study variables

Construct	*N*	Reliability and Validity		Model Fit Indicators						
		AVE	C.*R*	CFI	GFI	RMSEA	CMIN	DF	TLI	RMR
GAC	10	0.683	0.955	0.969	0.92	0.089	92.395	35	0.96	0.039
**GIC**										
GHC	12	0.567	0.928	0.852	0.742	0.092	1103.479	402	0.84	0.086
GSC	10	0.548	0.924							
GRC	8	0.577	0.915							
**SP**										
EP	4	0.526	0.916	0.916	0.886	0.089	201.14	76	0.883	0.052
SoP	6	0.509	0.878							
VP	5	0.505	0.834							
**GKM**										
GKA	5	0.512	0.839	0.842	0.747	0.095	822.682	289	0.823	0.06
GKS	5	0.519	0.843							
GKH	6	0.579	0.892							
GKP	5	0.452	0.803							
GKC	5	0.453	0.801							

Average Variances Extracted (AVE), Composite Reliability (C.R.), Root Mean Square Error of Approximation (RMSEA), root mean square residual (RMR), goodness-of-fit index (GFI), comparative fit index (CFI), Tacker-Lewis Index (TLI)

Table 4 presents the structural model results, demonstrating the direct and indirect effects of key variables on sustainable performance (SP). Green Absorptive Capacity (GAC) exhibited a statistically significant direct effect on SP (β = 0.123, *p* < 0.001), supporting H1. Green Intellectual Capital (GIC) also showed a significant direct effect on SP (β = 0.064, *p* = 0.016), supporting H2. Green Knowledge Management (GKM) had the strongest direct influence on SP (β = 0.727, *p* < 0.001), supporting H3. For the mediation effects, GKM significantly mediated the relationship between GIC and SP (β = 0.359, *p* < 0.001; supporting H4) and between GAC and SP (β = 0.256, *p* < 0.001; supporting H5). These findings underscore the importance of GKM in translating green competencies into sustainable leadership outcomes in nursing practice.

**Table 4:** Structural model results: hypothesis testing

Effects	Beta	t-value	*P*- value
GAC ---> SP	0.123	5.248	0.000
GIC ---> SP	0.064	2.409	0.016
GKM—> SP	0.727	15.088	0.000
GIC*GKM—> SP	0.359	8.179	0.000
GAC*GKM—> SP	0.256	6.377	0.000

**Fig. 2:**
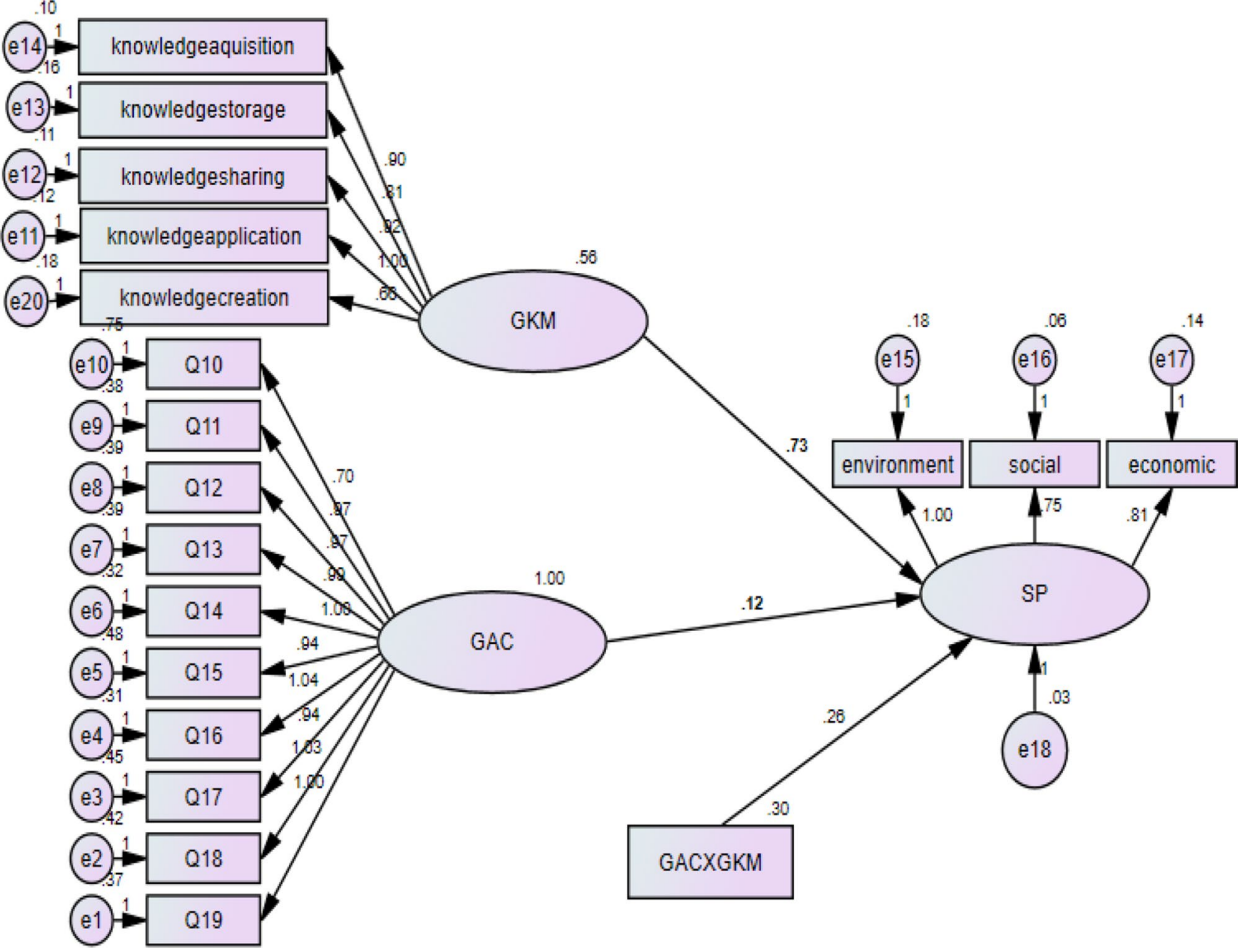
Structural model illustrating the mediating role of green knowledge management in the relationship between green absorptive capacity and sustainability performance. Notes: CMIN = 353.58, DF = 144, CMIN/DF = 2.455, CFI = 0.947, GFI = 0.846, TLI = 0.937, RMSEA = 0.084, RMR = 0.04

Figures 2 illustrates the structural equation model exploring the mediating role of Green Knowledge Management (GKM) in the relationships between Green Absorptive Capacity (GAC), Green Intellectual Capital (GIC), and Sustainable Performance (SP). The model confirms the strong direct influence of GKM on SP (β = 0.73), underscoring its pivotal role in driving sustainability in nursing leadership. The results validate the multidimensional nature of SP (environmental, social, and economic) and reinforce the importance of integrating green competencies into leadership strategies to promote sustainable healthcare systems.

Table 5 provides a concise overview of the study’s hypotheses, specifying each proposed relationship, the corresponding statistical results, and whether the hypothesis was supported.

**Table 5:** Summary of hypotheses testing results

Hypothesis	Statement	β	*p*-value	Support Status
H1	GAC → SP	0.123	< 0.001	Supported
H2	GIC → SP	0.064	0.016	Supported
H3	GKM → SP	0.727	< 0.001	Supported
H4	GIC → GKM → SP	0.359	< 0.001	Supported
H5	GAC → GKM → SP	0.256	< 0.001	Supported

## Discussion

Our study underscores a significant positive relationship between green absorptive capacity (GAC) and sustainable performance (SP) among nurse managers. This finding suggests that the ability of nurse managers to assimilate and apply green knowledge enhances sustainable performance by fostering environmentally responsible management practices. The integration of GAC into nursing leadership facilitates the development of sustainability-driven strategies, ultimately contributing to improved healthcare outcomes. These findings align with those of Zhou et al. (2023), who reported that GAC directly influences employees’ SP, and Ying, Hassan, and Afsar (2020), who emphasized that effectively managing GAC fosters knowledge creation and trust, thereby enhancing employees’ SP [42, 43]. However, unlike some earlier studies that focused primarily on industrial or manufacturing contexts, our results demonstrate that GAC has strong relevance in the healthcare sector, where sustainability involves complex patient care, regulatory compliance and resource optimization. This sector-specific evidence broadens the applicability of GAC theory and suggests that absorptive capacity in nursing leadership must account for the human-centered nature of healthcare delivery.

Regarding the role of green intellectual capital (GIC), findings indicate that it plays a pivotal role in enhancing SP, with green knowledge management (GKM) serving as a key mediator factor. Strengthening GIC requires adequate resources and effective knowledge management strategies, which collectively contribute to improved sustainability outcomes. Furthermore, GIC fosters collaboration and innovation, reinforcing its impact on sustainable performance within healthcare settings. These findings are consistent with previous research (Martín-Rubio, 2021; Sahoo et al., 2023), which established a positive association between GKM and GIC [8, 44]. Similarly, Haddad et al. (2024) emphasized that GIC acts as a bridge between external knowledge and internal organizational processes, facilitating sustainability [45]. By contrast, Nisar et al. (2021) argued that managing and developing green human capital primarily benefits stakeholders and enhances market value, with less emphasis on internal sustainability outcomes [46]. Our study challenges this view by showing that in nursing leadership, GIC not only supports external stakeholder value but also directly contributes to internal sustainable performance, likely due to the profession’s intrinsic service orientation and commitment to patient-centered care.

With regard to the influence of GKM, results indicate that an increase in GKM strengthens the impact of GAC on SP, emphasizing the importance of integrating technological and administrative green innovations in nursing leadership. By leveraging GKM, nurse managers can systematically embed sustainability principles into healthcare practices, optimizing environmental performance. These findings align with the work of Khan et al. (2024), who identified significant mediation effects and correlations between GKM and sustainable performance [9]. Similarly, Sahoo et al. (2022) established that GKM functions as a precursor to sustainability, reinforcing its critical role in advancing SP [47]. However, previous studies (Hsu & Sabherwal, 2012) have suggested that intellectual capital influences knowledge management in complex ways, warranting further investigation [48]. Our results refine this understanding by confirming a clear directional pathway in healthcare: GKM acts as an operational bridge that converts both GAC and GIC into tangible sustainability gains, indicating that in this context, the flow of influence is more unidirectional and implementation driven.

From a mediation perspective, the findings reveal that Green Knowledge Management (GKM) functions as a significant mediator between both Green Intellectual Capital (GIC) and Green Absorptive Capacity (GAC) in influencing Sustainable Performance (SP). This underscores GKM’s critical role in translating intangible organizational knowledge such as staff expertise, eco-innovation culture, and stakeholder collaboration into measurable sustainability outcomes. While Al-Hakimi et al. (2022) proposed GIC as a mediator between GKM and SP [49]. Our model contributes a novel inversion of this causal structure, positioning GKM as the mechanism through which GIC and GAC exert their influence on SP. This is aligned with Farzaneh et al. (2022), who emphasized the enabling role of intellectual capital in advancing sustainability [50]. Additionally, our results confirm that increased levels of GKM amplify the influence of GAC on SP, further supporting previous findings by Shahzad et al. (2020) and Xue et al. (2019), who identified GAC as a critical driver of sustainability in healthcare systems [51, 52]. This mediation evidence uniquely extends current nursing leadership literature by demonstrating that structured green knowledge systems are not merely supportive tools but essential mechanisms for operationalizing environmental and social sustainability goals in complex healthcare organizations.

To operationalize these findings, healthcare leaders and nurse managers should institutionalize GKM and GIC through targeted strategies. This may include the development of green leadership training programs that equip nursing managers with competencies in environmental decision-making, sustainability reporting, and climate-smart healthcare delivery. Moreover, establishing interdepartmental knowledge-sharing protocols such as digital knowledge repositories, green rounds, and peer exchange forums can promote the diffusion of green innovations across units. Hospitals may also benefit from creating eco-sustainability task forces, composed of cross-functional teams that assess current practices, implement green initiatives, and monitor environmental performance metrics. Embedding these strategies within organizational policy not only supports sustainable leadership but also aligns with broader Sustainable Development Goals (SDGs) in healthcare.

## Conclusion

This study provides empirical evidence on the pivotal role of green absorptive capacity and green intellectual capital in enhancing sustainable performance among nursing managers. The mediating effect of green knowledge management highlights the necessity of knowledge-driven sustainability strategies in healthcare leadership. Theoretically, the findings contribute to the emerging literature on environmental management in nursing by extending the application of resource-based and knowledge-based views within a healthcare context. Practically, the results underscore the importance of integrating environmental knowledge into nursing management to strengthen hospitals’ sustainability practices, reduce environmental footprints, and promote long-term organizational resilience. These findings call for targeted interventions to embed green competencies within nursing leadership, fostering a culture of sustainability in healthcare institutions.

### Implications of the study

The findings of this study carry several actionable implications for nursing management, healthcare policy, and leadership development. Nursing leadership programs should incorporate green absorptive capacity and intellectual capital to equip managers with tools for sustainability-driven decision-making. Educational curricula and in-service training should embed green knowledge management (GKM) competencies, focusing on eco-friendly workflows and environmental risk reduction. Policymakers are urged to implement governance frameworks that promote interdepartmental green knowledge sharing and sustainability audits. Additionally, healthcare systems should establish interdisciplinary sustainability committees that engage nurse managers in developing and overseeing eco-conscious strategies aligned with global health and environmental goals.

### Limitations of the study

While this study offers valuable insights into the interplay between green absorptive capacity, green intellectual capital, and green knowledge management in advancing sustainable performance among nursing managers, several limitations should be acknowledged. First, the cross-sectional design limits causal inference, as data were collected at a single point in time. This design prevents confirmation of the temporal order or directional nature of the observed relationships. Longitudinal research could provide stronger evidence by tracking changes in these constructs and their impact on sustainable performance over time. Second, reliance on self-reported questionnaires, although using validated tools, may have introduced response bias, such as social desirability or overestimation of environmental competencies and sustainability engagement. Future studies could mitigate this bias through triangulation, combining survey data with objective performance indicators, direct observations or qualitative interviews. Third, the study was conducted within a single healthcare system, potentially limiting the generalizability of the findings to different organizational structures, cultural contexts or healthcare delivery models. Multi-site, cross-cultural investigations would help verify the broader applicability of these results. Finally, the exclusive focus on managerial perspectives excluded frontline nurses, whose operational insights are crucial to understanding the full scope of sustainability implementation. Future research should adopt mixed-method approaches that integrate both strategic and operational viewpoints to provide a more comprehensive understanding of sustainability practices in nursing leadership.

## Data Availability

The datasets used and/or analyzed during this study are available from the corresponding author upon reasonable request.

## Abbreviations

GAC: Green Absorptive Capacity
GIC: Green Intellectual Capital
GKM: Green Knowledge Management
SP: Sustainable Performance
